# Prediction of problematic complexes from PPI networks: sparse, embedded, and small complexes

**DOI:** 10.1186/s13062-015-0067-4

**Published:** 2015-08-01

**Authors:** Chern Han Yong, Limsoon Wong

**Affiliations:** Graduate School for Integrative Sciences and Engineering, National University of Singapore, 28 Medical Drive, Singapore, 117456 Singapore; School of Computing, National University of Singapore, 13 Computing Dr, Singapore, 117417 Singapore

**Keywords:** Protein complex, Protein interaction, Data integration, Machine learning

## Abstract

**Background:**

The prediction of protein complexes from high-throughput protein-protein interaction (PPI) data remains an important challenge in bioinformatics. Three groups of complexes have been identified as problematic to discover. First, many complexes are sparsely connected in the PPI network, and do not form dense clusters that can be derived by clustering algorithms. Second, many complexes are embedded within highly-connected regions of the PPI network, which makes it difficult to accurately delimit their boundaries. Third, many complexes are small (composed of two or three distinct proteins), so that traditional topological markers such as density are ineffective.

**Results:**

We have previously proposed three approaches to address these challenges. First, Supervised Weighting of Composite Networks (SWC) integrates diverse data sources with supervised weighting, and successfully fills in missing co-complex edges in sparse complexes to allow them to be predicted. Second, network decomposition (DECOMP) splits the PPI network into spatially- and temporally-coherent subnetworks, allowing complexes embedded within highly-connected regions to be more clearly demarcated. Finally, Size-Specific Supervised Weighting (SSS) integrates diverse data sources with supervised learning to weight edges in a size-specific manner—of being in a small complex versus a large complex—and improves the prediction of small complexes. Here we integrate these three approaches into a single system. We test the integrated approach on the prediction of yeast and human complexes, and show that it outperforms SWC, DECOMP, or SSS when run individually, achieving the highest precision and recall levels.

**Conclusion:**

Three groups of protein complexes remain challenging to predict from PPI data: sparse complexes, embedded complexes, and small complexes. Our previous approaches have addressed each of these challenges individually, through data integration, PPI-network decomposition, and supervised learning. Here we integrate these approaches into a single complex-discovery system, which improves the prediction of all three types of challenging complexes. With our approach, protein complexes can be more accurately and comprehensively predicted, allowing a clearer elucidation of the modular machinery of the cell.

**Reviewers:**

This article was reviewed by Prof. Masanori Arita and Dr. Yang Liu (nominated by Prof. Charles DeLisi).

## Background

Protein complexes, which are stoichiometrically-stable structures consisting of multiple proteins that bind (interact) together, participate in many cellular processes. Determining the set of cellular complexes can help to elucidate the organization of cellular processes and their functional mechanisms.

Many approaches have been proposed to derive complexes from high-throughput protein-protein interaction (PPI) data, typically by searching for dense clusters in the PPI network that correspond to groups of interacting proteins. For example, Clique Finder (CFinder [1]) and Clustering by Maximal Cliques (CMC [2]) are based on finding and merging cliques; Algorithm for Identification of Protein Complexes (IPCA [3]) and ClusterONE [4] expand seeded vertices into clusters; Markov Clustering (MCL [5]) performs flow simulation to find dense partitions; Restricted Neighbourhood Search Clustering (RNSC [6]) modifies random clusterings to minimize a cost function. Some recent approaches have also incorporated prior biological knowledge, such as Coach [7], which models the core-attachment structure of many complexes.

In a previous paper [8] we described three challenges in complex prediction that arise from, or are exacerbated by, a static view of PPIs and protein complexes which are in fact dynamic in nature. First, many complexes are sparsely connected in the PPI network, and cannot be picked out by clustering algorithms which search for dense subgraphs. Second, many complexes are embedded within highly-connected regions of the PPI network with many extraneous edges connecting them to external proteins, so that clustering algorithms cannot properly delimit their boundaries. Third, many complexes are small (that is, composed of two or three proteins), making measures of important topological features, such as density, ineffectual.

We have proposed three approaches that can help to address these problems. First, Supervised Weighting of Composite Networks (SWC [9]) addresses the problem of sparse complexes. SWC integrates PPI data with two additional data sources, functional associations and co-occurrence in literature, and uses a supervised approach to weight edges with their posterior probabilities of belonging to a complex. SWC fills in the missing edges in many sparse complexes through data integration, and reduces the amount of spurious non-co-complex edges through supervised weighting. Using this approach, improvements are obtained in both precision and recall for yeast and human complex discovery, especially among the sparse complexes.

Second, decomposing PPI networks into spatially- and temporally-coherent subnetworks (DECOMP [10]) addresses the problem of complexes embedded in highly-connected regions with many extraneous edges. DECOMP removes hub proteins with large numbers of interaction partners, as they tend to correspond to date hubs with non-simultaneous interactions. Next, it decomposes the PPI network into spatially-coherent subnetworks using cellular-location Gene Ontology (GO) terms [11]. By splitting dense regions of the PPI network into less-dense but coherent subnetworks, complex-discovery performance is improved, with the biggest improvements among complexes embedded in highly-connected regions.

Third, Size-Specific Supervised Weighting (SSS [12]) addresses the problem of predicting small complexes. SSS integrates PPI data with two additional data sources, functional associations and co-occurrence in literature, along with their topological features, and uses a supervised approach to weight edges with their posterior probabilities of belonging to small complexes versus large complexes. SSS then extracts small complexes from the weighted network, and scores them using the probabilistic weights of edges within, as well as surrounding, the complexes. This approach achieves significant improvements in precision and recall in discovering small complexes.

**Fig. 1:**
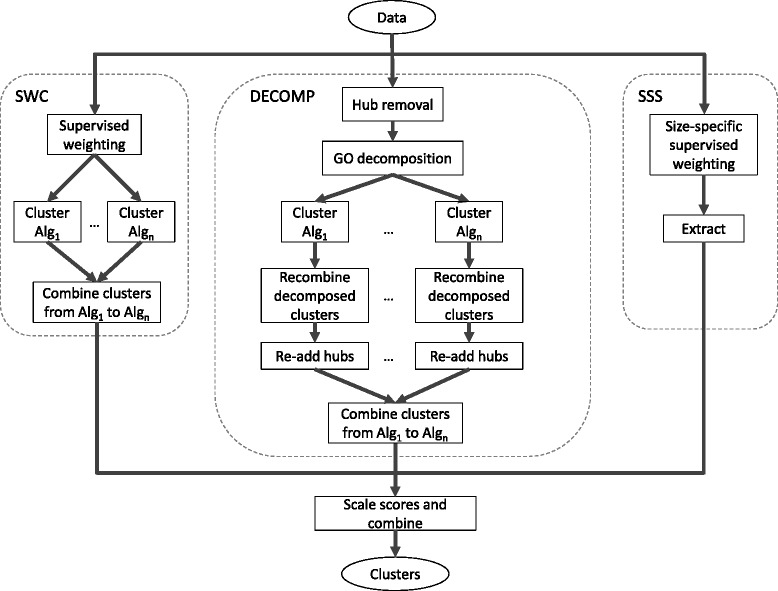
Flowchart of our integrated system consisting of Supervised Weighting of Composite Networks (SWC), PPI decomposition (DECOMP), and Size-Specific Supervised Weighting (SSS)

Although SWC and DECOMP both improve the prediction of large complexes in general, they have been shown to give the largest improvements among the complexes that they are designed for: sparse complexes for SWC, and complexes embedded in dense regions for DECOMP. The third technique, SSS, targets another separate group of complexes, the small complexes. Thus, here we combine these three techniques into a single system that targets all three groups of challenging complexes, as this is likely to give a performance boost in complex discovery over using any single one of these techniques. Figure 1 shows a flowchart of our integrated system consisting of SWC, DECOMP, and SSS. Each of these approaches is run independently on the input data, and the resulting clusters are combined at the end. Please refer to the Methods section for more details.

## Results and discussion

### Experimental setup

We compare the performance of the following approaches:

1. SWC+DECOMP+SSS: integrated approach consisting of SWC, DECOMP, and SSS
2. SWC: Supervised Weighting of Composite network, using six clustering algorithms combined with majority voting
3. DECOMP: decomposition of PPI network, using six clustering algorithms combined with majority voting
4. SSS: Size-Specific Supervised Weighting
5. PPI+COMBINE: PPI network weighted by reliability, using six clustering algorithms combined with majority voting
6. PPI+clustering algorithm: PPI network weighted by reliability, using a single clustering algorithm

We perform random sub-sampling cross-validation, repeated over ten rounds, using manually-curated complexes as reference complexes for training and testing. For yeast, we use the CYC2008 [13] set which consists of 408 complexes. For human, we use the CORUM [14] set which consists of 1829 complexes. Previously, we had tested our approaches on only large complexes (for SWC [9] and DECOMP [10]), or only small complexes (for SSS [12]); here, we test our integrated approach on all complexes, large and small. In each cross-validation round, *t%* of the complexes are selected for testing, while all the remaining complexes are used for training. Thus we use a large percentage of test complexes *t%*=90 *%*, giving 41 training complexes in yeast, and 183 training complexes in human. Each edge (*u*,*v*) in the network is given a class label *co-complex* if *u* and *v* are in the same training complex, otherwise its class label is *non-co-complex*. For SSS, the edges labeled *co-complex* are further split into two subclasses, *small-co-complex* and *large-co-complex*, for edges in small complexes (composed of two or three distinct proteins) and large complexes (composed of at least four distinct proteins), respectively. For the supervised approaches, learning is performed using these labels, and the edges of the entire network are then weighted using the learned models. The top-weighted *k* edges from the network are then used by the clustering algorithms to predict complexes. In our experiments we use *k*=20000 for SWC and DECOMP, and *k*=10000 for SSS (as described in their respective papers).

We use precision-recall graphs to evaluate how well the predicted clusters match the test complexes. Each cluster *P* is ranked by its score. To obtain a precision-recall graph, we calculate and plot the precision and recall of the predicted clusters at various cluster-score thresholds. Given a set of predicted clusters **P**={*P*_1_,*P*_2_,…}, a set of test reference complexes **C**={*C*_1_,*C*_2_,…}, and a set of training reference complexes **T**={*T*_1_,*T*_2_,…}, the recall and precision at score threshold *s* are defined as follows:

$$\begin{array}{c} Recall_{s} = \frac{\left| \{ C_{i} | C_{i} \in \textbf{C} \wedge \exists P_{j} \in \textbf{P},\, score(P_{j}) \geq s,\, P_{j} \; matches \; C_{i} \} \right|} {\left|\textbf{C}\right|} \end{array} $$

$$\begin{array}{l} Precision_{s} = \frac{\left| \{ P_{j} | P_{j} \in \textbf{P},\,score(P_{j}) \geq s \wedge \exists C_{i} \in \textbf{C}, C_{i} \, matches \, P_{j} \} \right|} {\left| \{ P_{k} | P_{k} \in \textbf{P},\, score(P_{k}) \geq s \wedge (\nexists T_{i} \in \textbf{T}, T_{i} \, matches \, P_{k} \vee \exists C_{i} \in \textbf{C}, C_{i} \; matches \, P_{k}) \} \right|} \end{array} $$

$$C \, matches \, P =\left\{ \begin{array}{ll} \text{true} & \text{if}\,\, size(C) > 3 \wedge size(P) > 3 \wedge Jaccard(P,C) \geq lg\_match \\ & \text{or}\,\, size(C) \leq 3 \wedge size(P) \leq 3 \wedge Jaccard(P,C) \geq sm\_match \\ \text{false} & \text{otherwise} \end{array} \right. $$

The precision of clusters is calculated only among those clusters that do not match a training complex, to eliminate the bias of the supervised approaches for predicting training complexes well. We require small complexes to be matched perfectly, as a mismatch of just one protein in a small complex may render the prediction less useful; on the other hand we allow a slight tolerance for mismatch for large complexes. Thus we require that small complexes must be matched by small clusters with a match threshold of *sm*_*match*, and large complexes must be matched by large clusters with a different threshold of *lg*_*match*. We define *lg*_*match*=0.75 for large yeast complexes, *lg*_*match*=0.5 for large human complexes (since they are more challenging to predict), and *sm*_*match*=1 for small complexes in both yeast and human.

### Complex prediction

**Fig. 2:**
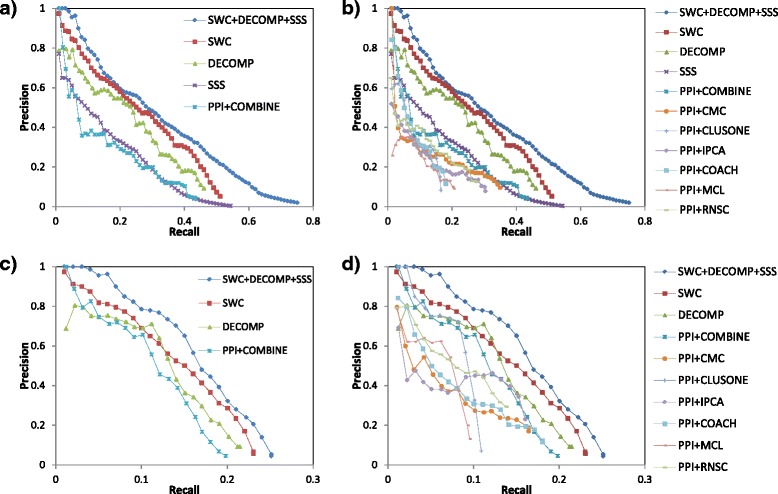
Precision-recall graphs for complex prediction in yeast. For clarity, **a** shows only the integrated approach (SWC+DECOMP+SSS), each of its constituent approaches, and the PPI+COMBINE approach, while **b** includes the individual clustering algorithms. **c** and **d** show the performance when the generated small clusters are removed, which lowers recall substantially but increases precision. In yeast we use a matching threshold of *l* *g*_*m* *a* *t* *c* *h*=0.75 for large complexes, and *s* *m*_*m* *a* *t* *c* *h*=1 for small complexes

Figure 2 shows the precision-recall graphs for complex prediction in yeast. Figure 2a shows that SWC and DECOMP both attain higher precision than PPI+COMBINE, demonstrating the benefits of supervised weighting and PPI decomposition (note that all three of these approaches use the COMBINE strategy). As SSS’ predictions are limited to small complexes, which is moreover a difficult challenge with a perfect matching requirement, it has lower precision levels compared to PPI+COMBINE. However, the integrated approach, SWC+DECOMP+SSS, is able to predict both large and small complexes, and achieves much higher recall as well as precision. Figure 2b shows that individual clustering algorithms (used with the PPI network) give lower precision and recall compared to PPI+COMBINE, showing the utility of combining the clusters from multiple clustering algorithms.

 We noticed that the generated small clusters may depress the precision, as many of them are false positives. Figures 2c and d show the performance when these small clusters are removed. As expected, recall drops substantially, as the small complexes are now unable to be predicted: for example, for PPI+COMBINE, recall drops from over 40 *%* to about 20 *%*. However, precision is improved, as the many false-positive small clusters are removed. For our integrated approach(SWC+DECOMP+SSS), the removal of small clusters means removing those clusters generated by SSS. We still achieve higher precision and recall than the other approaches, showing that our integrated approach still outperforms other approaches when considering large complexes only. Moreover, *without* removing small clusters, our integrated approach maintains high precision as it uses a specialized approach, SSS, to predict small complexes.

**Fig. 3:**
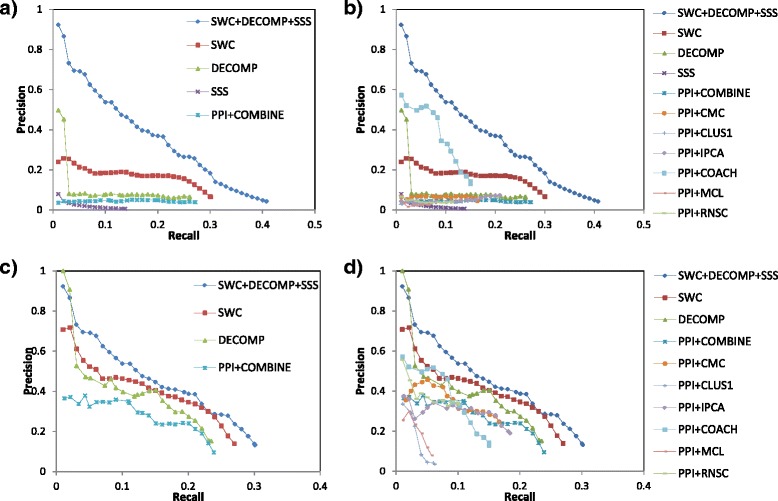
Precision-recall graphs for complex prediction in human. For clarity, **a** shows only the integrated approach (SWC+DECOMP+SSS), each of its constituent approaches, and the PPI+COMBINE approach, while **b** includes the individual clustering algorithms. **c** and **d** show the performance when the generated small clusters are removed, which lowers recall but increases precision. For human we use a matching threshold of *l* *g*_*m* *a* *t* *c* *h*=0.5 for large complexes, and *s* *m*_*m* *a* *t* *c* *h*=1 for small complexes

Figure 3 shows the corresponding precision-recall graphs for complex prediction in human. Figure 3a shows that SWC and DECOMP both attain higher precision than PPI+COMBINE, showing the benefits of supervised weighting and PPI decomposition. SSS shows poor performance as it is limited to predicting small complexes, which is especially challenging in human. The integrated approach, SWC+DECOMP+SSS, is able to predict both large and small complexes, and achieves higher recall as well as precision. Figure 3b shows that most of the individual clustering algorithms (used with the PPI network) give lower precision or recall compared to PPI+COMBINE, showing the utility of combining the clusters from multiple clustering algorithms. The exception is Coach, which attains high precision as it does not generate small clusters by design, thereby cutting down on its false-positive predictions.

Figures 3c and d show the performance when the generated small clusters are removed. Compared to yeast, here the recall does not drop as much: for example, for PPI+COMBINE, recall drops by about 5 *%* only. However, the improvement in precision is substantial: for example, PPI+COMBINE sees more than fivefold increase in precision at many points in the graph. This reveals an issue in complex prediction which is more obvious in human but still apparent in yeast: predicting small complexes alongside large ones means accepting a drop in precision due to large numbers of false-positive small clusters; while improving precision by excluding small clusters means that no small complexes can be predicted. On the other hand, our integrated approach uses a specialized approach, SSS, to generate the small clusters separately from the large ones, which allows effective prediction of the small complexes while still maintaining high precision levels.

**Fig. 4:**
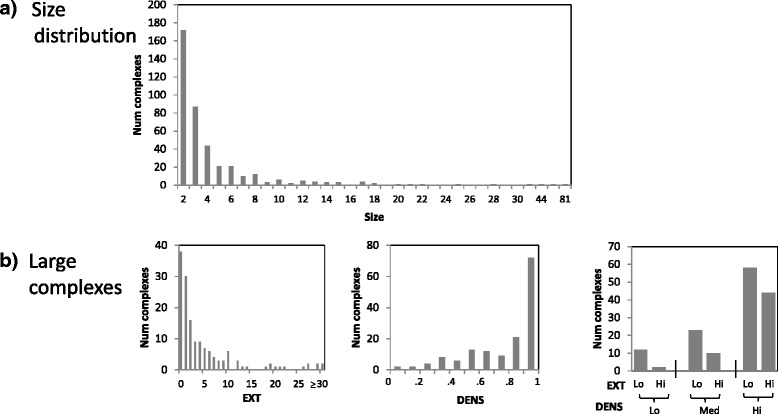
Statistics of yeast reference complexes

**Fig. 5:**
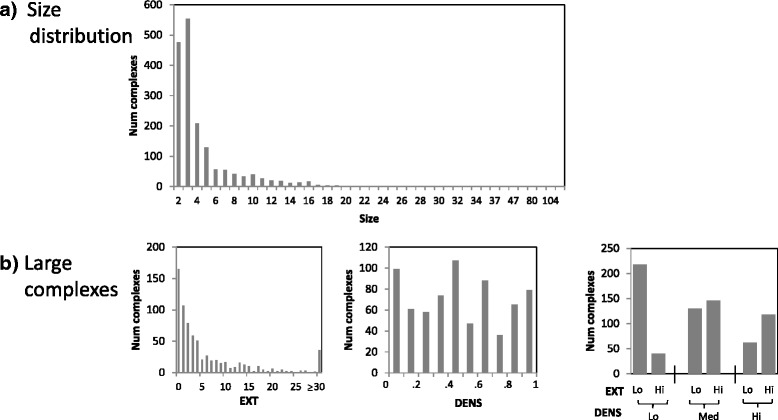
Statistics of human reference complexes

To investigate the performance of our integrated approach with respect to the three challenges that we highlighted, we stratify the reference complexes in terms of their sizes, extraneous edges, and densities. First, to quantify whether a complex is embedded within a highly-connected region of the PPI network, we derive EXT, the number of external proteins that are highly connected to it, defined as being connected to at least half of the proteins in the complex. Second, to quantify how sparse a complex is, we derive DENS, the density of each complex, defined as the number of PPI edges in the complex divided by the total number of possible edges in the complex. In our analysis, we stratify the complexes into large and small complexes, and further stratify the large complexes into low, medium, and high DENS (corresponding to DENS of [ 0,.35], (.35,.7], and (.7,1] respectively), and low and high EXT (corresponding to EXT ≤3 and >3 respectively), to give seven total strata (one for small complexes, and six for large complexes). Figures 4 and 5 show the size distribution, and the DENS and EXT analysis strata of the large complexes, of the yeast and human complexes. Note that the less-challenging complexes to predict are the large complexes with high DENS and low EXT, and these correspond to around only 15 *%* and 5 *%* of complexes in yeast and human respectively; the remaining complexes are challenging in some way, with the majority of them falling into the small-complex category.

**Fig. 6:**
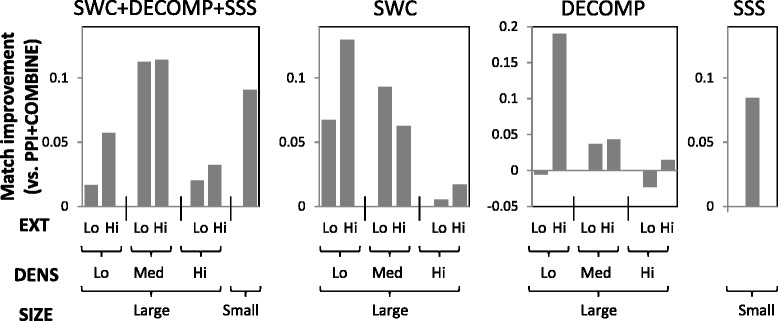
Match-score improvements among stratified yeast complexes

**Fig. 7:**
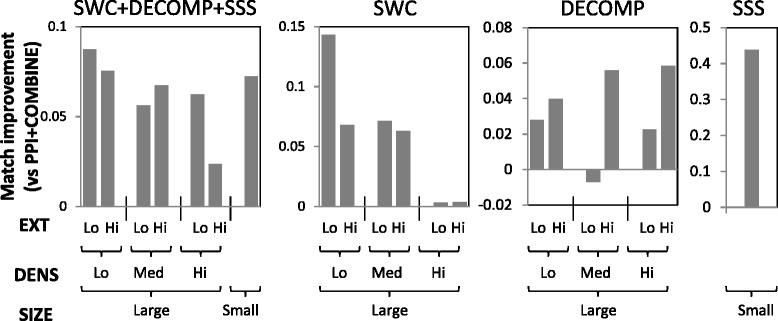
Match-score improvements among stratified human complexes

We take the top 1000 clusters generated by each approach, and determine how well the reference complexes in the different strata are matched by these clusters. Figures 6 and 7 show the average improvements in matching scores among the stratified complexes for our approaches versus PPI+COMBINE, in yeast and human respectively.

Among yeast and human large complexes, SWC gives the biggest improvements among complexes with low to medium density: it uses data integration and supervised learning to fill in missing edges of sparse complexes to allow them to be predicted. Among sparse complexes, even those with high EXT see an improvement, showing that SWC’s supervised weighting can effectively reduce the number of spurious edges in the PPI network. DECOMP gives the biggest improvements among complexes with high EXT, within each density stratum. This is because it decomposes the PPI network into spatially- and temporally-coherent subnetworks, in which complexes may become disconnected from their original densely-connected neighbourhoods, allowing their borders to be better delimited by clustering algorithms. As expected, SSS improves the performance among small complexes. Our integrated approach (SWC+DECOMP+SSS) spreads out the improvements among the complexes in the different strata, showing that the different approaches complement each other to predict different types of challenging complexes.

### Novel complexes

Here we investigate the number and quality of novel complexes predicted by our approaches. For the supervised approaches, we use the entire sets of reference complexes for training. We keep only predicted complexes that are novel, unique, and high-confidence. First, predicted complexes that are similar to each other are filtered to keep only the highest-scoring one. Next, we keep only the top-scoring predictions such that the precision of these predictions (i.e. proportion of predictions that match a reference complex) is greater than 0.4. Finally, we keep only novel predictions by removing those that match a reference complex. We use a Jaccard similarity threshold of 0.5 in the above procedure for matching.

We measure the quality of these novel predictions by their semantic coherence in each of the three GO classes, biological process (BP), cellular compartment (CC), and molecular function (MF). First, we use the most informative common ancestor method to calculate the semantic similarity between two GO terms [15]. Then we define the semantic coherence between two proteins as the highest semantic similarity between their two sets of annotated GO terms, for each GO class. Finally the semantic coherence of a set of proteins is their averaged pairwise semantic similarity, for each GO class.

**Fig. 8:**
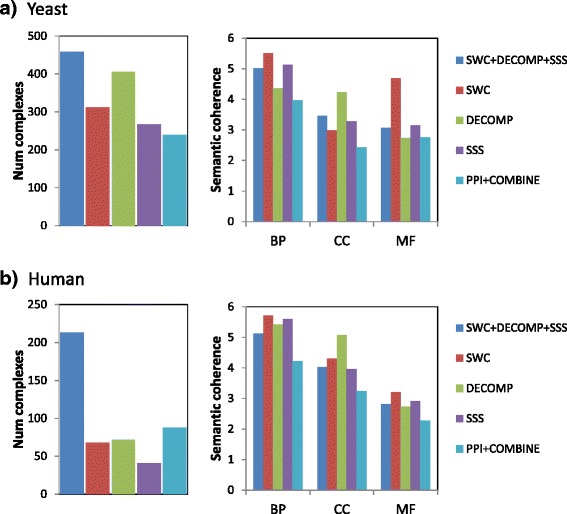
Number and quality of novel predictions in **a** yeast, **b** human

Figure 8a shows the number and quality of novel predictions in yeast. Each of our individual approaches (SWC, DECOMP, and SSS) predicts more novel complexes compared to the baseline (PPI+COMBINE), while the integrated approach generates the highest number of novel complexes. The novel complexes from our individual approaches attain higher semantic coherence in one or more of the GO classes, compared to the baseline. The novel predictions from the integrated approach attain semantic coherence that is averaged out between its three constituent approaches, which gives it higher coherence than the baseline across all three GO classes.

Figure 8b shows the number and quality of novel predictions in human. As described above, PPI+COMBINE generates a great number of small clusters in human, most of which are false-positives; this gives it a greater number of novel predictions compared to each of our individual approaches. Nonetheless, our integrated approach still generates the greatest number of novel complexes. As in yeast, our individual approaches generate novel complexes with greater semantic coherence compared to PPI+COMBINE; the integrated approach achieves greater semantic coherence, in all three GO classes, in its predictions compared to the baseline. Thus, in both yeast and human, our integrated approach generates the greatest number of novel predictions, with higher quality compared to the baseline approach of combined clustering with a PPI network.

An example novel human complex discovered by our integrated approach consists of proteins PELP1, SENP3, TEX10, and LASIL1. This complex is not part of our reference complexes, but is validated as four out of five members of a recently-characterized 5FMC complex [16]. This complex is also predicted by SWC and DECOMP at low scores (ranked 103 and 12 respectively); through voting, our integrated approach re-scores it much higher (second-highest rank).

## Conclusion

Three open problems remain within protein-complex prediction. First, many complexes are sparsely connected in the PPI network, and so do not form dense clusters that can be derived by clustering algorithms. Second, many complexes are embedded within highly-connected regions of the PPI network, which makes it difficult for clustering algorithms to accurately delimit their boundaries. Third, many complexes are small (composed of two or three distinct proteins), so that traditional topological markers such as density or sparse boundaries are ineffective.

Previously, we proposed three approaches for addressing each of these challenges. First, Supervised Weighting of Composite Networks (SWC) integrates diverse data sources with supervised learning to weight edges with their posterior probabilities of being co-complex. SWC was shown to improve the prediction of sparse complexes. Next, PPI network decomposition using GO terms and hub removal (DECOMP) was shown to improve the prediction of complexes embedded within highly-connected regions. Finally, Size-Specific Supervised Weighting (SSS) integrates diverse data sources and topological features with supervised weighting to weight edges with their posterior probabilities of belonging to small complexes. SSS was shown to improve the prediction of small complexes.

Here we integrate these three approaches into a single system. SWC, DECOMP, and SSS are run independently on the input PPI data and other data sources, and the resulting clusters are scaled to standardize their scores, then combined using majority voting. We test the integrated approach on the prediction of yeast and human complexes, and show that it outperforms SWC, DECOMP, or SSS when run individually, achieving the highest recall, and the highest precision at all recall levels.

We also investigate which complexes benefit most from our individual approaches and the integrated approach, compared to a baseline of running a set of clustering algorithms on a reliability-weighted PPI network. In both yeast and human, we find that SWC improves the prediction of sparse complexes, DECOMP improves the prediction of embedded complexes, and SSS improves the prediction of small complexes. The integrated approach combines these improvements and distributes them among the different types of challenging complexes.

Although we have taken great strides in tackling the three challenges we highlight within complex prediction, and have obtained substantial improvements in prediction accuracy and recall as a result, there remains room for further improvement. Moreover, as increasing amounts of PPI data become available for other organisms, the techniques that we propose will be useful in enabling the discovery of novel complexes in those organisms.

The integrated complex-prediction software package and data files are available at http://compbio.ddns.comp.nus.edu.sg/~cherny/IntegratedComplexPrediction/.

## Methods

In this section we describe how we integrate our three techniques, SWC, DECOMP, and SSS, into a single system. We first describe the data sources and clustering algorithms used, then describe the integrated system.

### Data sources and features

Our three approaches use different sets of data features (although SSS uses of all of them). These features are derived from three different data sources—PPI, functional association, and literature co-occurrence—and their topological characteristics. Each data source provides a list of scored protein pairs: for each pair of proteins (*a*,*b*) with score *s*, *a* is related to *b* with score *s*, according to that data source. For both yeast and human, the following data sources are used:

- *PPI*: We take the union of physical PPIs from three databases: BioGRID [17], IntAct [18] and MINT [19] (data from all three repositories downloaded in January 2014). In yeast we additionally incorporate the Consolidated PPI dataset [20]. The PPIs are scored using a simple reliability metric. First, we estimate the reliability of each experimental detection method *e* as the fraction of its detected PPIs where both interaction partners share at least one high-level cellular-component Gene Ontology term. Then the reliability of an interaction (*a*,*b*) is estimated as:
  $$\begin{array}{@{}rcl@{}} reliability(a,b) = 1 - \prod\limits_{e \in E_{a,b}} (1 - rel_{e})^{n_{e,a,b}} \end{array} $$
  where *rel*_*e*_ is the estimated reliability of experimental method *e*, *E*_*a*,*b*_ is the set of experimental methods that detected interaction (*a*,*b*), and *n*_*e*,*a*,*b*_ is the number of times that experimental method *e* detected interaction (*a*,*b*). The scores from the Consolidated dataset are discretized into ten equally-spaced bins (0−0.1,0.1−0.2,…), each of which is considered as a separate experimental method in our scoring scheme. We avoid duplicate counting of evidences across the datasets by using their publication IDs (in particular, PPIs from the Consolidated dataset are removed from the BioGRID, IntAct, and MINT datasets).
- *STRING*: STRING predicts functional associations between proteins using various evidence types, including genetic context and experimental data [21] (data downloaded in January 2014). Each evidence type is given a confidence score based on co-occurrence in KEGG pathways, and the scores from various evidence types are combined to give a final functional-association score for each protein pair. Only pairs with scores greater than 0.5 are kept.
- *LIT*: We calculate the co-occurrence of proteins or genes in PubMed literature (data downloaded in June 2012), using the Jaccard similarity of the sets of papers that a pair (*a*,*b*) appears in:
  $$\begin{array}{@{}rcl@{}} s = \frac{\left|A_{a} \cap A_{b}\right|} {\left|A_{a} \cup A_{b}\right|} \end{array} $$
  where *A*_*x*_ is the set of PubMed papers that contain protein *x*. For yeast, that would be the papers that contain the gene name or open reading frame (ORF) ID of *x* as well as the word “cerevisiae”; for human that would be the papers that contain the gene name or Uniprot ID of *x* as well as the words “human” or “sapiens”.

For each protein pair in each data source, we derive three topological features: degree (DEG), shared neighbors (SHARED), and neighborhood connectivity (NBC). For each data source, the edge weight used to calculate these topological features is the data source score of the edge.

- *DEG*: The degree of the protein pair (*a*,*b*), or the sum of the scores of the outgoing edges from the pair:
  $$\begin{array}{@{}rcl@{}} DEG(a,b) = \sum\limits_{x \in N_{a} \setminus \{b\}} w(a,x) + \sum\limits_{x \in N_{b} \setminus \{a\}} w(b,x) \end{array} $$
  where *w*(*x*,*y*) is the data source score of edge (*x*,*y*), *N*_*a*_ is the set of all neighbours of *a*, excluding *a*.
- *NBC*: The neighborhood connectivity of the protein pair (*a*,*b*), defined as the weighted density of all neighbors of the protein pair excluding the pair themselves:
  $$\begin{array}{@{}rcl@{}} NBC(a,b) = \frac{\sum\limits_{x,y \in N_{a,b}} w(x,y)} { min\left(\left| N_{a,b} \right|,\lambda\right) \left(min(\left| N_{a,b} \right|, \lambda) - 1\right)} \end{array} $$
  where *w*(*x*,*y*) is the data source score of edge (*x*,*y*); *N*_*a*,*b*_ is the set of all neighbours of *a* and *b*, excluding *a* and *b* themselves; *λ* is a dampening factor.
- *SHARED*: The extent of shared neighbors between the protein pair, derived using the Iterative AdjustCD function (with two iterations) [2].

A final topological feature, isolatedness (*ISO(a,b)*), represents the probability that the protein pair (*a*,*b*) is in a size-2 or size-3 clique which is isolated from the rest of the network. It is derived from an initial calculation of the small-co-complex posterior probabilities (see [12] for details).

Table 1 lists the features used in each of the three approaches. DECOMP makes use of only the PPI feature, as the incorporation of additional edges from additional features may exacerbate the problem of embedded complexes which it addresses. SWC further incorporates additional edges from STRING and LIT, as well as a topological PPI feature, to help fill out the edges within sparse complexes, while the other features are unnecessary for this purpose. Finally, SSS further includes all the remaining topological features, as they help to distinguish true small complexes from false positives.

**Table 1:** Data used for our three approaches

Feature	Description	SWC	DECOMP	SSS
*P* *P* *I*	PPI reliability	✓	✓	✓
*STRING*	Functional association	✓		✓
*LIT*	Literature co-occurrence	✓		✓
*D* *E* *G*_*PPI*_	PPI topological degree			✓
*D* *E* *G*_*STRING*_	STRING topological degree			✓
*D* *E* *G*_*LIT*_	LIT topological degree			✓
*S* *H* *A* *R* *E* *D*_*PPI*_	PPI shared neighbours	✓		✓
*S* *H* *A* *R* *E* *D*_*STRING*_	STRING shared neighbours			✓
*S* *H* *A* *R* *E* *D*_*LIT*_	LIT shared neighbours			✓
*N* *B* *C*_*PPI*_	PPI neighbourhood connectivity			✓
*N* *B* *C*_*STRING*_	STRING neighbourhood connectivity			✓
*N* *B* *C*_*LIT*_	LIT neighbourhood connectivity			✓
*ISO*	Isolatedness			✓

### Clustering algorithms

We use the following clustering algorithms in our approach:

**Markov Cluster Algorithm (MCL)** [5] simulates flow through a network to find regions with high flow bounded by regions with lower flow. It converges to a partition of highly-connected regions (the clusters) separated by sparse boundaries.

**Restricted Neighborhood Search Clustering (RNSC)** [6] starts from an initial random clustering, and moves nodes between clusters to minimize a cost function based on the number of intra- and inter-cluster edges.

**IPCA** [3] starts from seeded vertices and expands them into clusters by incorporating new vertices, based on the connectivity between the vertices and clusters, as well as the diameters of the resultant clusters.

**Clustering by Maximal Cliques (CMC)** [2] first generates maximal cliques from a network. Cliques that overlap highly are merged if they are highly inter-connected, otherwise the clique with lower weighted density is removed.

**Clustering with Overlapping Neighborhood Expansion (ClusterONE)** [4] starts from seeded vertices, and expands them to maximize a cohesiveness function based on the edge weights both within and surrounding a cluster. Highly-overlapping clusters are merged.

**Coach** [7] adopts the core-attachment model of complexes to detect complexes in two stages: core detection, and complex formation. First, cores are found as vertices that have higher-than-average local degree in their neighbourhood subgraphs, and whose induced neighbourhoods are dense. Next, proteins that are connected to at least some proportion of each core’s vertices are recruited as attachments to the core.

SWC and DECOMP both use a simple voting-based aggregative strategy, called COMBINE, to take the union of the clusters produced by multiple clustering algorithms. If two or more clusters are found to be similar to each other, then only the cluster with the highest weighted density is kept, and its score is its weighted density multiplied by the number of algorithms that produced the group of similar clusters; otherwise its score is its weighted density as usual. The cluster scores are then normalized to a maximum of 1 by dividing by the number of clustering algorithms used. We define two clusters *C* and *D* to be similar if *Jaccard*(*C*,*D*)≥0.75, where *Jaccard*(*C*,*D*) is the Jaccard similarity between the proteins contained in *C* and *D*:

$$Jaccard(C, D) = \frac{\left|V_{C} \cap V_{D}\right|} {\left|V_{C} \cup V_{D}\right|} $$

where *V*_*X*_ is the set of proteins contained in *X*.

### Integrated complex-prediction system

Figure 1 shows a flowchart of our integrated system. SWC, DECOMP, and SSS are run independently on the input data, and the resulting clusters are combined. Here we give only a brief description of each approach, and leave the reader to refer to the original publications for the details; where there are any differences with the originally-described approaches, we also mention them here.

First, SWC [9] integrates its four input data (see Table 1), using supervised learning with a maximum-likelihood naive-Bayes model to weight each edge with its posterior probability of being a co-complex edge. Then it runs the various clustering algorithms, and combines the resulting cluster sets with majority voting to produce its set of clusters. Each final cluster is scored by its weighted density (weights being the SWC posterior probabilities), multiplied by the number of clustering algorithms that produced it, and normalized to 1. We keep only clusters of size four or larger. Note that the original SWC approach (as described in [9]) used only three features (*SHARED*_*PPI*_, *STRING*, and *LIT*); here we incorporate the *PPI* feature, as the reliability of a PPI is an effective indicator of a co-complex relationship.

Next, DECOMP [10] performs hub removal on its input PPI data, by removing proteins with more than *N*_*hub*_ interaction partners (where *N*_*hub*_=50 for yeast, *N*_*hub*_=150 for human; these parameters, and others in DECOMP, are derived experimentally as described in [10]). Then it performs GO decomposition to split the PPI network into spatially-coherent subnetworks, where each subnetwork is the induced PPI subnetwork of proteins annotated to a cellular-component GO term. The GO terms used for decomposition are chosen as those terms annotated to at least *N*_*GO*_ proteins, while none of their descendents (in the GO ontology) are annotated to more than *N*_*GO*_ proteins (where *N*_*GO*_=300 for yeast, *N*_*GO*_=1000 for human). For each of the clustering algorithms, the algorithm is run on the subnetworks, the clusters from the subnetworks are re-combined, and hubs are re-added to those clusters they are highly-connected to. Finally, the resulting clusters from the various clustering algorithms are combined with majority voting to produce a set of clusters. Each cluster is scored by its weighted density (weights being the PPI reliabilities), multiplied by the number of clustering algorithms that produced it, and normalized to 1. We keep only clusters of size four or larger. Note that the original PPI decomposition approach (as described in [10]) did not aggregate clusters from multiple clustering algorithms, but used individual clustering algorithms instead; here we incorporate the COMBINE strategy for DECOMP as it was found to improve complex-discovery performance in [9]. The original approach also used only the BioGRID PPI dataset, whereas here we further integrate the IntAct and MINT PPI datasets.

Next, SSS [12] integrates its thirteen input data (see Table 1), using supervised learning with a maximum-likelihood naive-Bayes model to weight each edge in a size-specific manner: each edge is weighted with its small-co-complex, large-co-complex, and non-complex posterior probabilities. Then the small complexes (size-2 and -3 complexes) are extracted, and scored by cohesiveness-weighted density, which takes into account the weights of both internal and surrounding edges.

Finally, the clusters produced by the three approaches are combined, also using the voting-based aggregative strategy. However, since each approach scores its clusters in a different manner, we first scale their scores to make them comparable. The clusters generated by DECOMP are scaled by a factor *d*, while those generated by SSS are scaled by a factor *s* (clusters generated by SWC are implicitly scaled by a factor of 1.0). In our experiments we used *d*=0.6,*s*=1.0 for yeast, and *d*=0.6,*s*=0.3 for human. These factors were obtained by observing the relationship between scores and precision levels in the cross-validation results for each approach (e.g. a cluster predicted by DECOMP with a score of 0.6 obtained roughly the same precision as a cluster predicted by SWC with a score of 1.0). Then we take the union of the clusters produced by the three approaches. If a cluster from two or more approaches are found to be similar to each other (Jaccard similarity ≥0.75), we sum its scores from the different approaches.

## Reviewers’ comments

### Reviewer’s report 1

Prof. Masanori Arita, National Institute of Genetics, Japan

The manuscript describes the summarizing work of the authors’ previous three published results. The current work is an integration or meta-tool approach by dissecting the given PPI dataset by complex sizes and other characters.

1. BMC Syst Biol. 2014, 8(Suppl 5):S3
2. BMC Syst Biol. 2012;6 Suppl 2:S13
3. Proteome Sci. 2011 Oct 14;9 Suppl 1:S15.

Overall, the approach taken is sound and the results are well justified. The flow of the manuscript is, however, extremely similar to their 2nd work in 2012. There is no paragraph copied or identical but essentially, methods and assessments are the same. This in part inevitable because the approach taken is similar. Still, to emphasize novelty, I would like to encourage authors to add more insights obtained by this meta approach. In fact, their paper in 2012 introduces more detailed analysis on each complex identified. Along this line, suggested idea is focusing on some human or yeast specific protein complexes compares their function with the previous work in 2012. what I could not fully understand was the slightly different use of STRING database. STRING is in itself a meta-tool for PPI, and was used in the previous approach too (2012 paper). Is its usage different this time? Then what is the reason?

### Authors’ response:

Indeed, the main contribution of this paper is to integrate our previously-presented complex-prediction methods into a single tool, rather than to propose an entirely new complex-prediction method. Since this work integrates the 3 previous methods, it does not predict any novel complexes that were not predicted by any of the 3 methods. Instead, we show that the power and accuracy of the integrated approach is much improved, especially for human complexes. Nevertheless we have revised the paper to add an example validated complex (5FMC) which was low-scoring using the individual approaches, but now become higher-scoring after integrating the 3 approaches together (due to the voting-based scoring).

The STRING database is used in the same way in SWC in the 2012 paper and in SWC in the current paper: as a datasource for functional associations, not necessarily PPIs (this is the consequence of how STRING scores its associations). So we consider the STRING data as functional associations to be quite orthogonal to our PPI data. The SSS method further includes additional topological features derived from STRING: the degree, neighbourhood connectivity, and shared neighbours, in the STRING network. This is because these features are useful in distinguishing true small complexes from false positives: while many size-2 and size-3 groups of proteins form isolated clusters in the PPI network by chance, it is less likely that they also form isolated clusters in the STRING network and the LIT network. We have revised the paper to describe the use of features in the different methods.

### Reviewer’s report 2

Dr. Yang Liu (nominated by Prof. Charles DeLisi), Boston University, USA

This paper integrates three approaches that they identified before into a single system to predict problematic complexes. They apply the system to both yeast and human PPI networks and can achieve higher accuracy and more comprehensive prediction, compared with three individual methods.

Major:

1. In Background, instead of only introducing their own work, the authors may also consider talking about other similar studies to predict complexes, especially for recent years.
2. It seems that methods proposed in this paper are not parameter-free, not only for three individual ones, but also for the integrated approach. There are too many parameters to fix, like N_*hub*, d, s etc. Without any explanation, it makes readers confused about how to choose these parameters to get the best performance. Are they based on experimental tests, or some statistical calculation? The authors may need to demonstrate how they make the selection.
3. The authors claim that integrated method outperforms three individual methods, which seems obvious because those three methods try to address three different challenges, and an integrated version could no doubt work better. Have the authors tried to compare their method to other independent complexes prediction approach?

### Authors’ response:

1. We have revised the Background section to include a brief mention of some popular complex-discovery algorithms. Some of these are further described in the Clustering algorithms subsection of the Methods section, as these are the algorithms that we make use of and which we compare our performance against.
2. Parameters specific to the individual approaches, like N_*hub* and N_*go*, were determined experimentally in their original papers; we have modified our paper to clarify this. The parameters specific to the integration approach are d and s: these are scaling parameters to calibrate the scores of DECOMP and SSS, and their recommended values have been found through cross-validation experiments.
3. Indeed, Figure 2b show the precision-recall graphs for yeast complex prediction, using our approaches (integrated approach and the three individual approaches), as well as six popular complex-prediction algorithms: CMC, ClusterOne, IPCA, Coach, and MCL. Figure 3b show the corresponding graphs for human complex prediction. Our approaches outperform these other approaches. Figures 2d and 3d also show that these approaches are hampered by trying to predict small complexes: when their predicted small clusters are removed, their precisions increase substantially; nonetheless even here our approaches still outperform them.

## Abbreviations

PPI: Protein-protein interaction
SWC: Supervised Weighting of Composite Networks
DECOMP: PPI-network decomposition
SSS: Size-Specific Supervised Weighting
CMC: Clustering by Maximal Cliques
IPCA: Algorithm for Identification of Protein Complexes
RNSC: Restricted Neighbourhood Search Clustering
GO: Gene Ontology
EXT: Number of external proteins highly-connected to complex
DENS: Complex density
DEG: Degree
SHARED: Shared neighbours
NBC: Neighbourhood connectivity
ISO: Isolatedness
